# Thoracic Back Pain Leading to an Acromegaly Diagnosis: A Case Report

**DOI:** 10.7759/cureus.85533

**Published:** 2025-06-07

**Authors:** Ammar Siddiqui, Melissa Chao, Michael Rahimi, Alla Spivak, Michael Weinberger

**Affiliations:** 1 Anesthesiology, New York Medical College, Valhalla, USA; 2 Anesthesiology, Columbia University, New York, USA

**Keywords:** acromegaly, back pain, diffuse idiopathic skeletal hyperostosis (dish), growth hormone, mid back pain

## Abstract

Diffuse idiopathic skeletal hyperostosis (DISH), a common cause of thoracic backache, may be caused by unregulated growth factor activity by increasing enthesis and bony growth. Excess growth factors can cause acromegaly. We describe the case of a patient presenting with chronic thoracic axial back pain for several decades and features of acromegaly on history and physical exam. Single photon emission computed tomography (SPECT) CT was obtained to identify the pain generator and detected increased metabolic activity in the anterior column suggestive of DISH. Subsequent laboratory testing revealed acromegaly. Follow-up imaging identified a growth hormone (GH)-secreting pituitary macroadenoma with local invasion. To the best of our knowledge, this is the first time the finding of DISH has hinted at the possibility of acromegaly, and may illustrate the need for added vigilance for acromegaly in patients with DISH.

## Introduction

Diffuse idiopathic skeletal hyperostosis (DISH) is characterized by calcification and ossification of ligaments and entheses, particularly the anterior longitudinal ligament. It affects over 25% of individuals in some populations [1]. Although its etiology is unclear, anabolic growth factors such as growth hormone (GH) and insulin-like growth factor-1 (IGF-1) are implicated in its development [2,3]. Hyperostosis of the spine was originally believed to be due to heavy lifting and thoracic kyphosis but more recent studies have shown a primary metabolic etiology. 

Acromegaly is a systemic endocrine disorder caused by chronic GH excess, typically due to a pituitary adenoma [4]. The global prevalence is estimated at 60 per million people [5], though delayed diagnosis is common due to the gradual onset of symptoms and the normalization of physical changes [6].

Given the overlap in the pathophysiology of DISH and acromegaly, we propose that DISH (especially in conjunction with physical signs such as macroglossia and acral enlargement) may serve as an early, objective radiologic clue to acromegaly. Prior studies have investigated the role of hyperinsulinemia and metabolic factors between acromegaly and DISH [7] but, to our knowledge, this is the first case of acromegaly diagnosis with DISH as the first radiologic clue. Pain clinics, where imaging is often first initiated for back pain evaluation, are in a unique position to recognize such associations. Differentials of midback pain can include lumbar spondylosis, discogenic pain, and infectious causes such as discitis. Single photon emission computed tomography (SPECT) CT imaging can be a useful tool to differentiate between these disorders are areas of inflammation will show greater avidity, signaling them as potential pain generators.

This report adheres to the EQUATOR and CARE guidelines. Ethics approval was obtained from the New York Medical College institutional review board. Written informed consent was provided by the patient. The authors declare no conflicts of interest or funding sources.

## Case presentation

A 65-year-old male construction supervisor presented to the New York Medical College pain clinic with persistent mid-thoracic axial back pain for over one year. The pain was dull, non-radiating, and worsened with axial loading. Nonsteroidal anti-inflammatory drugs (NSAIDs) and physical therapy were ineffective.

His medical history included type 2 diabetes mellitus, hypertension, and obstructive sleep apnea. He noted recent changes including macroglossia and increased ring tightness. Family history was negative for endocrine disorders.

Physical examination revealed coarse facial features, prognathism, widely spaced teeth, and acral enlargement. He was 6'5" (196 cm), 114 kg (BMI: 30 kg/m^2).

**Figure 1:**
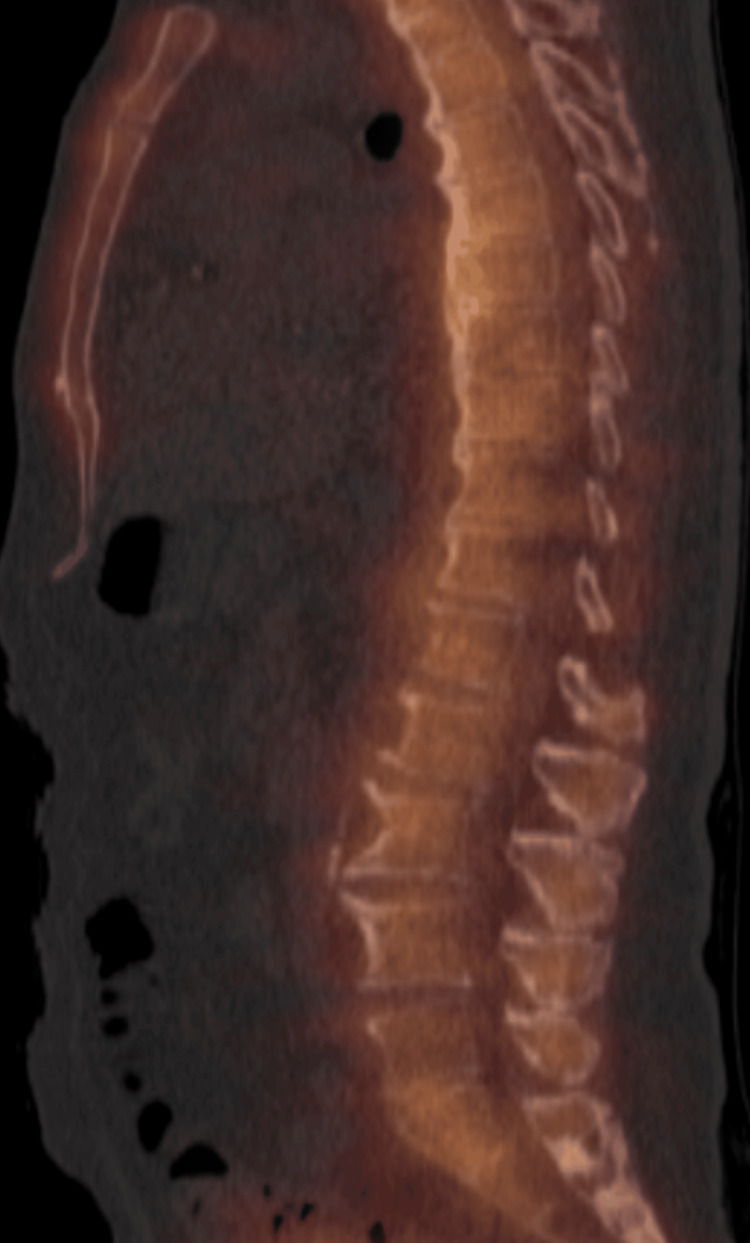
SPECT CT sagittal cut showing diffusely increase uptake along the anterior thoracic spine, reflecting DISH SPECT CT: single-photon emission computed tomography and computed tomography, DISH: diffuse idiopathic skeletal hyperostosis

Thoracic spine MRI showed mild disc protrusions without spinal canal stenosis. A SPECT CT scan demonstrated increased radiotracer uptake along the anterior margin of T12 and L1 and the thoracic spine, consistent with DISH (Figure 1).

These findings prompted hormonal testing. IGF-1 was elevated at 227 ng/mL (repeat: 239 ng/mL). GH levels failed to suppress on oral glucose tolerance testing (nadir GH: 2.05 ng/mL).

**Figure 2:**
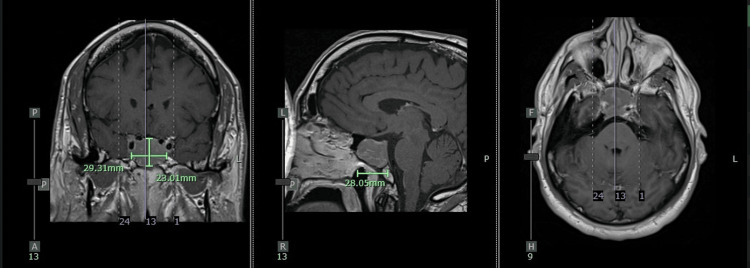
T1 post contrast MRI Brain showing pituitary macroadenoma invading right internal carotid artery (left sagittal cut, middle coronal cut, right axial cut)

Subsequent brain MRI identified a 2.9 x 2.8 x 2.3 cm pituitary macroadenoma invading the right cavernous sinus and encasing the right internal carotid artery, with extension into the sphenoid sinus (Figure 2).

The patient underwent trans-sphenoidal resection of the adenoma. Immunohistochemistry confirmed GH-producing tumor cells. Postoperative endocrine follow-up and medical therapy were initiated.

## Discussion

This case highlights the diagnostic value of recognizing systemic features of acromegaly in patients presenting to pain clinics. Thoracic back pain accounts for a significant proportion of clinic visits [8], and when imaging reveals DISH, it should prompt consideration of systemic causes such as GH excess.

Up to 75% of acromegaly patients may develop hyperostotic skeletal changes [9]. While acral and facial changes are well-known signs, they are often overlooked or misattributed. DISH, however, provides an objective imaging finding that can initiate an endocrine evaluation.

Differential diagnoses for chronic thoracic back pain include mechanical strain, vertebral fracture, metastatic disease, ankylosing spondylitis, and infection. When standard workup is unrevealing and DISH is present, a metabolic or endocrine disorder should be considered.

Early recognition of acromegaly is critical. Surgical remission rates for pituitary microadenomas approach 80%, while macroadenomas have lower remission rates (<30%) [10]. As pain specialists often order initial imaging and assess for neuropathic symptoms like carpal tunnel syndrome, they are well-positioned to detect signs of acromegaly.

Finally, while MRIs of the lumbar spine are very sensitive, SPECT CTs are more specific and can sometimes identify the pain generator and inflammation better than MRIs. They come with a cost of greater radiation to the patient but can be considered if the diagnosis remains elusive.

## Conclusions

To the best of our knowledge, we report the first known case of DISH prompting evaluation for and diagnosis of acromegaly. This underscores the potential for pain clinics to identify systemic diseases through imaging and physical examination. Increasing awareness of endocrine signs among pain physicians may facilitate earlier diagnosis and better outcomes.

## References

[1] Fournier DE, Leung AE, Battié MC, Séguin CA (2024). Prevalence of diffuse idiopathic skeletal hyperostosis (DISH) and early-phase DISH across the lifespan of an American population. Rheumatology (Oxford).

[2] Sarzi-Puttini P, Atzeni F (2004). New developments in our understanding of DISH (diffuse idiopathic skeletal hyperostosis). Curr Opin Rheumatol.

[3] Denko CW, Boja B, Malemud CJ (2002). Growth hormone and insulin-like growth factor-I in symptomatic and asymptomatic patients with diffuse idiopathic skeletal hyperostosis (DISH). Front Biosci.

[4] Fernandez A, Karavitaki N, Wass JA (2010). Prevalence of pituitary adenomas: a community-based, cross-sectional study in Banbury (Oxfordshire, UK). Clin Endocrinol (Oxf).

[5] Crisafulli S, Luxi N, Sultana J (2021). Global epidemiology of acromegaly: a systematic review and meta-analysis. Eur J Endocrinol.

[6] Dekkers OM, Biermasz NR, Pereira AM, Romijn JA, Vandenbroucke JP (2008). Mortality in acromegaly: a metaanalysis. J Clin Endocrinol Metab.

[7] Littlejohn GO, Hall S, Brand CA, Davidson A (1986). New bone formation in acromegaly: pathogenetic implications for diffuse idiopathic skeletal hyperostosis. Clin Exp Rheumatol.

[8] Halfpap J, Riebel L, Tognoni A, Coller M, Sheu RG, Rosenthal MD (2022). Improving access and decreasing healthcare utilization for patients with acute spine pain: five-year results of a direct access clinic. Mil Med.

[9] Lugo G, Pena L, Cordido F (2012). Clinical manifestations and diagnosis of acromegaly. Int J Endocrinol.

[10] Swearingen B, Barker FG 2nd, Katznelson L (1998). Long-term mortality after transsphenoidal surgery and adjunctive therapy for acromegaly. J Clin Endocrinol Metab.

